# Case Report: Next Generation Sequencing in Clinical Practice–A Real Tool for Ending the Protracted Diagnostic Odyssey

**DOI:** 10.3389/fcvm.2021.778961

**Published:** 2022-01-13

**Authors:** Alena S. Limonova, Alexandra I. Ershova, Alexey N. Meshkov, Anna V. Kiseleva, Mikhail G. Divashuk, Marina V. Kurkina, Oxana M. Drapkina

**Affiliations:** ^1^Laboratory of Clinomics, National Medical Research Center for Therapy and Preventive Medicine of the Ministry of Healthcare of the Russian Federation, Moscow, Russia; ^2^Laboratory of Molecular Genetics, National Medical Research Center for Therapy and Preventive Medicine of the Ministry of Healthcare of the Russian Federation, Moscow, Russia; ^3^Kurchatov Genomics Center-ARRIAB, All-Russia Research Institute of Agricultural Biotechnology, Moscow, Russia; ^4^Laboratory of Inherited Metabolic Diseases, Federal State Budgetary Scientific Institution “Medical Genetic Scientific Center Named After Academician N.P. Bochkova”, Moscow, Russia; ^5^Department of Fundamental and Applied Aspects of Obesity, National Medical Research Center for Therapy and Preventive Medicine of the Ministry of Healthcare of the Russian Federation, Moscow, Russia

**Keywords:** genetic testing, next generation sequencing, *ABCG8*, sitosterolemia, macrothrombocytopenia, premature coronary artery disease

## Abstract

We reported a case of sitosterolemia, which is a rare genetic disease, characterized by increased plant sterol absorption and great heterogeneity of clinical manifestations. Our patient was initially referred to the lipid clinic due to high cholesterol levels and premature cardiovascular disease. Diagnosis of familial hypercholesterolemia was established in accordance with the Dutch Lipid Clinic Network criteria. Next-generation sequencing was later performed, which revealed a nonsense mutation in the *ABCG8* gene, which led to the diagnosis of sitosterolemia. The aim of our report is to demonstrate, how genetic testing helped to make the correct diagnosis and to explain many of the patient's health problems, which etiology remained unclear for many years.

## Introduction

Despite almost the same consumption of cholesterol and plant sterols with the western diet, in comparison with cholesterol, only trace amounts (~5%) of plant sterols are absorbed, with further quick excretion into the bile from hepatocytes (1). Disruption of these mechanisms by defective ABCG5/ABCG8 leads to the accumulation of plant sterols in body tissues. This genetic disorder, sitosterolemia (MIM 210250), is a rare autosomal recessive disease, first described in 1974 (2). It is caused by mutations in either of ATP binding cassette (ABC) transporter genes (*ABCG5* and *ABCG8*). Although recent data suggest that not only homozygous but also heterozygous carriers of *ABCG5* loss-of-function variants exhibit increased plant sterols and LDL-C levels and have increased risk of coronary artery disease (CAD), compared with non-carriers (3). These proteins are expressed only in hepatocytes, gallbladder epithelium, and enterocytes and function as obligate heterodimers responsible for excretion of sterols, with plant sterols preferred over cholesterol (4, 5). Thus, they perform a basic physiological process for the prevention of the accumulation of dietary plant sterols. Deficiency of *ABCG5* or *ABCG8* in sitosterolemia impairs excretion of plant sterol into the intestinal lumen from enterocytes and into bile in the liver, thereby resulting in a severe accumulation of plant sterols in plasma and tissues.

In this report, we presented a case of a patient with sitosterolemia and discuss mechanisms of different clinical manifestations of the disease, present in our patient. We aimed to demonstrate how next-generation sequencing (NGS) helped to make the correct diagnosis, which explained all the symptoms, present in this patient and which etiology remained unclear for many years.

## Case Description

A 41-year-old woman was referred to the lipid clinic due to her elevated cholesterol level. On examination, she had tendinous xanthomas of the Achilles tendons. Her body weight was 59 kg, and her height was 1.67 m, with a body mass index of 21.2 kg/m^2^. She had exertional dyspnea since adolescence. Hypercholesterolemia was revealed at the age of 34 years old (total cholesterol – 10.12 mmol/L, LDL-C – 8.8 mmol/L, HDL-C – not available). Two years later, due to the onset of angina and ischemic changes on ECG during physical activity, coronary arteriography was performed. It revealed 80% stenosis of the left main coronary artery, 80% stenosis of the anterior descending artery, occlusions of the proximal right coronary artery, the diagonal branch, and the obtuse marginal branch. At that time the duplex sonography revealed no atherosclerotic manifestations of other vascular territories (carotids and lower extremity arteries). Coronary artery bypass grafting (CABG) was performed. During the first 2 days after CABG, the patient had hemopericardium and hemothorax. She was diagnosed with intracerebral hemorrhage 10 days later.

On rosuvastatin 20 mg and ezetimibe 10 mg, which were initiated 1 year before the current presentation, her total cholesterol was 6.7 mmol/L, LDL-C - 3.95 mmol/L, HDL-C – 2.3 mmol/L. During follow-up with a cardiologist paroxysmal atrial fibrillation was diagnosed. Three years after CABG, the stress testing was performed with positive results. One year later, the CT-angiography revealed subtotal stenosis of the left main coronary artery, with normally functioning bypass grafts to the left anterior descending artery and right coronary artery. Duplex sonography demonstrated stenosis of carotid bifurcations and proximal internal carotid arteries up to 30% at the right and 35% at the left, and 35% stenosis of the left vertebral artery.

Her medical history was also significant for macrothrombocytopenia (with current PLT 113 x 10^*^9/L and MPV 14.7 fL), arthralgia of knee joints, and past recurrent serositis (pericarditis and pleurisy). The latter was treated with fluoroquinolones and prednisolone. Cardiolipin antibodies and anti-dsDNA test results were negative. No definite diagnosis was established. Due to the polycystic ovary syndrome, the resection of ovaries was fulfilled at the age of 23. Her gynecologic history was also noteworthy for frozen pregnancy of unknown etiology, weak labor activity which led to caesarian section (she has one child, a boy with no clinical manifestations), and early menopause at 39 years old.

Her family history was unremarkable with no information about premature CAD.

According to the Dutch Lipid Clinic Network criteria (16 points) heterozygous familial hypercholesterolemia (HeFH) was diagnosed. Maximum statin dose, ezetimibe, and proprotein convertase subtilisin/kexin type 9 (PCSK9) inhibitors were prescribed and cascade screening was recommended.

Later on, the NGS was performed using the Ion S5 (Thermo Fisher Scientific, USA). Ampliseq libraries were prepared on the Ion Chef System (Thermo Fisher Scientific, USA) using the custom panel, created with the Ion AmpliSeq Designer (Thermo Fisher Scientific, USA), which consists of 25 genes (*ABCA1, ABCG5, ABCG8, ANGPTL3, APOA1, APOA5, APOB, APOC2, APOC3, APOE, CETP, GPD1, GPIHBP1, LCAT, LDLR, LDLRAP1, LIPC, LIPI, LMF1, LPL, MTTP, PCSK9, SAR1B, STAP1, USF1*) and 280 variants responsible for the lipid metabolism.

**Figure 1:**
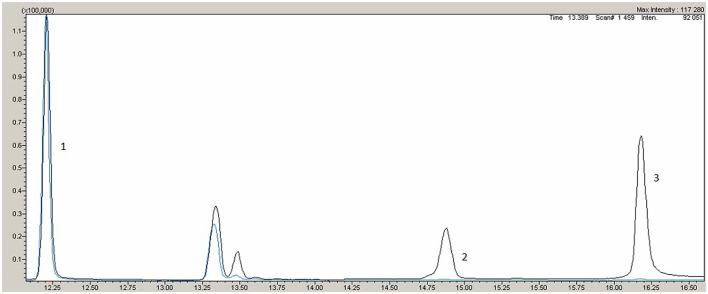
Gas chromatography-mass spectrometry. Black line - patient, blue line – normal reference. 1- internal standart, 2 - campresterol, 3 – sitosterol.

Variants with total frequency <0.01 identified in the patient are listed in Table 1. Missense variants in heterozygous state in the cholesteryl ester transfer protein (*CETP)* (NM_000078.2: c.708G>C, p.Gly236Arg) and apolipoprotein B *(ApoB)* (NM_000384.2: c12382G>A, p.Val4128Met) genes were revealed, wherein both were classified as variants of uncertain significance according to the American College of Medical Genetics and Genomics guidelines (ACMG2015). Pathogenic or likely pathogenic variants in *LDLR, PCSK9, APOB*, and *LDLRAP1* were not detected. The patient was found to be homozygous for a nonsense mutation in the *ABCG8* gene (NM_022437.2: c.1083G > A, p.Trp361Ter), leading to premature termination of signal codon 361 (6). According to ACMG2015 this variant is classified as pathogenic (criteria PVS1, PP5, PM2, PP3) (7). These variants were confirmed by Sanger sequencing on 3,500 DNA Analyzer (Thermo Fisher Scientific, USA). A multiplex sitosterol assay for diagnosis of sitosterolemia was performed by gas chromatography-mass spectrometry (GC-MS) TQ-8050 (Shimadzu, Japan) with autosampler AOC-20i, HP-5MS. Sample preparation and analysis conditions were performed with GC-MS according to Joon Hee Lee et al. (8). Her plant sterol levels in blood were markedly elevated: sitosterol 54.4 μmol/L, ref. 0.4 – 3.4 μmol/L; campesterol 18.8 μmol/L, ref.0.1 – 3.1 μmol/L (Figure 1), confirming the diagnosis. The limitation of the current work is no genetic and biochemical data being available from the relatives (with them residing too far away) and lack of follow-up data from the patients, as she refused further medical evaluation because of the COVID-19 pandemic.

**Table 1:** List of variants identified in the patient.

**Gene**	***APOB***	***ABCG8***	***CETP***
Genomic coordinates (GRCh37)	chr2:21225912	chr2:44099233	chr16:57005951
Transcript	NM_000384.2	NM_022437.2	NM_000078.2
cDNA change	c.12382G>A	c.1083G>A	c.706G>C
Heterozygous/ Homozygous	Heterozygous	Homozygous	Heterozygous
dbSNP	rs1801703	rs137852987	Not applicable
Kind of mutation	Missense	Nonsense	Missense
SIFT/ PolyPhen	Tolerated/ benign	-	Tolerated/ benign
Protein Change	p.Val4128Met	p.Trp361Ter	p.Gly236Arg
GnomAD total allele frequency	0.000004608	0.0009374	Not described
ACMG	VUS	Pathogenic	VUS

*Non-synonymous substitutions of coding regions and variants in the canonical splice sites with minor allele frequency <0.01 (according to the Genome Aggregation Database (gnomAD), https://gnomad.broadinstitute.org/, 06 September 2021;) were included in the table. ACMG, American College of Medical Genetics and Genomics; VUS, variant of unknown significance. APOB, apolipoprotein B; ABCG8, ATP-binding cassette G8; CETP, cholesteryl ester transfer protein*.

According to current clinical guidelines, for very high cardiovascular risk patients with atherosclerotic CAD target LDL-C level is <1.4 mmol/L, thus the patient was prescribed with maximum statin dose, ezetimibe, PCSK9 inhibitors. Plant sterol-free diet was recommended with restriction of the consumption of shellfish, margarine, vegetable oils, nuts, seeds, avocados, chocolate, whole grains, olives. Genetic testing of the patient's son was recommended. As it was revealed during the follow-up visit, the patient took statin and ezetimibe, with poor adherence to the sterol-free diet.

## Discussion

Sitosterolemia is a rare genetic disease. To date, approximately 110 homozygous individuals with sitosterolemia have been reported (9). For rare diseases, an average pathway to correct diagnosis takes 7.3 years mean, with 7 physicians and 2–3 misdiagnoses during the patient's diagnostic odyssey (https://globalgenes.org/rare-disease-facts/). This odyssey is, undoubtedly, a real burden for patients suffering from misdiagnoses and inappropriate treatment and for the healthcare system. For example, as described by Wang et al. in their series of cases, the mean ages of symptoms onset were 15.2 years, and the mean years of delay between symptom onset and diagnosis were 28.8 (range 15–49 years) (10). For our patient, it lasted for 7 years.

However, sitosterolemia may be not so rare, as previously believed. Evidence from recent research demonstrates, that misdiagnosis of sitosterolemia with familial hypercholesterolemia may be prevalent (11, 12), which is also the case of our patient. The great variety of possible manifestations means that physicians of different specialties, like cardiologists, hematologists, and rheumatologists can deal with this disease. But even in the case of a competent specialist who will have a suspicion of this rare disorder, the diagnosis needs confirmation. We experienced a real challenge in search of GC-MS laboratory, as it is usually used in research but not clinical ones. In the case of our patient, NGS helped to make the correct diagnosis.

Sitosterolemia is characterized by great clinical heterogeneity. This could be explained by a large variety of xenosterols present in human diets which may differ in their biological activity, but only a few sterols are commonly detected in the studies [details discussed in (13)]. Furthermore, we would like to discuss clinical manifestations present in our patients in order to demonstrate how the correct diagnosis, revealed by NGS, could at once explain many of the patient's different conditions, which could not be correctly diagnosed and treated previously.

### Hypercholesterolemia

Patients with sitosterolemia may present with normal cholesterol levels, as in the first described cases of the disease (2). However, some sitosterolemic patients have elevated cholesterol levels, as in this case and some previously described ones (14, 15). One of the possible explanations is that deletion in *ABCG8* reduces biliary cholesterol secretion (16), and excretion of sterols into the bile seems pivotal for their accumulation, as liver transplantation in the patient with sitosterolemia led to complete normalization of the plant sterols levels (17). The turnover rates of plasma cholesterol and plant sterols in patients with sitosterolemia were similar, meaning increased retention and reduced elimination of both plant sterols and cholesterol, as was demonstrated by Salen et al. (18) with the usage of *in vivo* radiolabeled isotopic techniques. Some bias may come from the inability of conventional clinical methods to discriminate between cholesterol and plant sterols as these methods depend on the C-5 double bond or the presence of the 3β hydroxyl group, both of which are present in cholesterol and plant sterols (19). High cholesterol levels are more characteristic for children with their further tendency to decrease. For instance, Mymin et al. (20) revealed an inverse association between age and plasma cholesterol. However, for understanding the precise mechanisms of hypercholesterolemia further research is needed, as till now, there is more evidence for normal/reduced cholesterol levels. Thus, in hepatic tissues of affected individuals increased LDL receptors activity alongside reduced activity of the rate-limiting enzyme for cholesterol biosynthesis (3 -hydroxy-3- methyl-glutaryl-CoA reductase) was demonstrated (21). Yu et al. (22) showed that ABCG5/ABCG8-deficient mice had higher plant sterols levels in their plasma and liver, and had reduced cholesterol synthesis relative to wild-type mice. Results of the studies addressing the molecular mechanisms, by which plant sterols influence cholesterol homeostasis, proposed that plant sterols may not only compete with dietary and biliary cholesterol for intestinal absorption in mixed micelles but also interfere with different steps of cholesterol metabolism, i.e., cholesterol esterification and lipoprotein assembly, cholesterol internalization, cholesterol synthesis and removal of apoB100-containing lipoproteins [details are reviewed by Calpe-Berdiel et al. (23)].

The missense variant in the *CETP* gene revealed in our patient (NM_000078.2: c.708G>C, p.Gly236Arg) is not described in gnomAD, where another substitution (c.708G>A, p.Gly236Ser) was previously described with total frequency <0.01. SNPs, reported at http://www.hgmd.cf.ac.uk/ as associated with decreased CETP activity/increased HDL-C, are often located at beta strands within the protein sequence. Two hundred and thirty six amino acid position is also located at the beta-strand region. Thus, the patient's phenotype (HDL-C - 2.3 mmol/L) could be explained by this missense variant.

### Xanthomas, Atherosclerosis, and Cardiovascular Disease

Clinical data regarding atherosclerosis development vary from early myocardial infarction and sudden cardiac death at the age of 5 (24) to no clinical manifestations (15), even among family members of an affected individual (20). Premature CAD may develop even in the case of normal cholesterol levels (25, 26).

The same clinical heterogeneity is true about xanthomas (20, 27). Our patient manifested with xanthomas of the Achilles tendons and premature premature CAD (marked atherosclerotic damage of coronary arteries diagnosed at the age of 36).

From enterocytes plant sterols, as well as cholesterol esters, proceed to chylomicrons which are further metabolized in the liver. Lipoprotein-derived cholesterol and plant sterols can further penetrate the artery wall and lead to inflammation and atherosclerotic plaque progression (28). In case of their high level in the blood, plant sterols can be found in atherosclerotic lesions and xanthomas (2, 29). Enhanced accumulation of sterols (including plant sterols) in homozygous individuals can accelerate foam cells formation (30). Plant sterols seem to have more cytotoxic effects, than cholesterol (31). However, different plant sterols demonstrated opposite results on inflammatory cytokine secretion in cultured macrophage foam cells (32). Bao et al. (33) proposed one of the possible explanations of how plant sterols can promote atherosclerosis progression. Authors demonstrated that macrophages incubated with sitosterol-containing lipoproteins undergo death in an accelerated manner, compared with free-cholesterol-induced macrophages death. As macrophage death is a key event for plaque disruption (34, 35), such data provide at least one mechanism for accelerated atherothrombosis in patients with very high levels of plant sterols (33). Plant sterols are more susceptible to oxidative processes than cholesterol, though further research is needed to demonstrate the health effects of their oxidized forms (36).

Similar mechanisms could be involved in xanthomas formation, as early stages of their formation resemble early stages of atherogenesis (37). Moreover, a lower plasma level of plant sterols is needed for xanthomas formation in comparison with cholesterol level (38).

In a recent genetic study, based on data from biobanks of Iceland, Denmark, and UK Biobank, authors evaluated the association of variants in *ABCG5/ABCG8* with non-HDL cholesterol, a plant sterols, and the risk of CAD. They demonstrated that the degree of CAD risk conferred by *ABCG5/ABCG8* variants is greater than predicted by their effect on non-HDL cholesterol levels only. The authors concluded that plant sterols may contribute to atherogenesis directly, irrespective of non-HDL cholesterol (39). Koeijvoets et al. (40) studied the association between two polymorphisms in the *ABCG8* gene and CAD in 2012 patients with heterozygous familial hypercholesterolemia. They demonstrated that individuals carrying the risk genotype for both *ABCG8* variants had an increased risk of cardiovascular disease (RR 1.57, 95% CI 1.13–2.18; *p* = 0.01) and coronary heart disease (RR 1.72, 95% CI 1.23–2.41; *p* = 0.002). Wu et al. (41) investigated the association between four *ABCG5* and *ABCG8* SNPs and CAD. Binary logistic regression analysis demonstrated that the homozygous C allele at Thr400LysC>A (rs4148217) resulted in a more than 2-fold greater risk of developing CAD, compared with those who carried the A allele in a dominant model. The multivariate analysis supported that such an effect was independent of several other risk factors, including age, gender, history of diabetes mellitus, and HDL-C level.

### Polycystosis

Our patient has a compromised gynecological anamnesis. As complete medical records of that period were unavailable, we can't definitely conclude, whether or not her gynecological anamnesis could be related to her main disease or some other problem. Anyway, we suppose that this aspect of her health problems is worth mentioning and discussing in light of high plant sterol levels.

Pieces of evidence from some animal studies and case reports of this disease suggest that high plant sterol levels may interfere with endocrine function and fertility. For instance, a family including three siblings, homozygous for sitosterolaemia, with adrenal insufficiency and ovarian failure was described previously (42). Solca et al. (43) demonstrated, that *ABCG5/ABCG8* knock-out mice fed with a diet high in plant sterols were infertile. Exclusion of plant sterols from diet or blockage of their absorption (with ezetimibe) could restore fertility. No structural abnormalities of the ovaries were found. Oxidation products of plant sterols have been reported to modulate the action of 17β-estradiol *in vitro* and two human cell lines (44). In addition, plant sterols lowered plasma estrogen levels in mice (45). In double knock-out *ABCG5/ABCG8* mice adrenal glands had a 91% reduction in cholesterol content, treatment with ezetimibe returned adrenal cholesterol levels to near-normal levels, however, the response of the glands to ACTH was unimpaired (46).

### Macrothrombocytopenia

Our patient has both macrothrombocytopenia and bleeding abnormalities. The association of stomatocytic hemolysis and macrothrombocytopenia with sitosterolemia was revealed by Rees et al. In five pedigrees with these hematologic abnormalities they demonstrated the presence of both elevated levels of plant sterols in blood and mutations in *ABCG5* and *ABCG8*, previously linked to sitosterolemia (47). Sitosterolemia may manifest solely with hematologic problems (48). *In vitro* experiments showed changes in shape and osmotic fragility of red blood cells when incubated in the presence of sitosterol (48).

Thrombocytopenia caused by sitosterolemia may be confused with other causes, including autoimmune disorders, which leads to unnecessary diagnostic steps and inappropriate steroid treatment (49). And in our patient autoimmune disorders were also ruled out previously. In one recent observational study among seven patients with unexplained long-standing thrombocytopenia, whole-exome sequencing helped to reveal sitosterolemia in one of them. It remains unclear why only some individuals develop hematologic manifestations of the disease. Further research is needed to find out whether other genetic/environmental factors contribute. But among the variants that have been reported to cause sitosterolemia, only 24 have been associated with macrothrombocytopenia with the variant found in our patient among the most prevalent ones (50).

Since ABCG5 and ABCG8 are not present on the surfaces of red blood cells and platelets, insertion of plant sterols into their membranes is supposed to be the most likely explanation. Kruit et al. (51) in experiments with *ABCG5*-deficient mice demonstrated, that they still had macrothrombocytopenia after transplantation of bone marrow from wild-type mice. At the same time, the bone marrow transplantation from *ABCG5*-deficient mice to wild-type mice did not result in platelet changes. Research by Kanaji et al. (52) improved our understanding of mechanisms, contributing to bleeding abnormalities and macrothrombocytopenia. It was demonstrated, that accumulation of plant sterols in platelet plasma membranes leads to their hyperactivity and that internalization of the αIIbβ3 complex and filamin A degradation cause macrothrombocytopenia and bleeding phenotype (52).

### Arthralgia

Arthralgia is another possible clinical manifestation of sitosterolemia (10, 53), with subsiding severity of the symptom with treatment (53). Arthralgia of knee joints is also present in our patient. To our knowledge, data regarding the mechanism of this clinical symptom seem to be missing in the literature. Togo et al. (53) proposed, that the accumulation of plant sterols in immune cells in sitosterolemia patients modulates the immune system.

## Conclusion

Sitosterolemia is a rare genetic disease with great heterogeneity of clinical manifestations. Many of them were present in our patient but their real etiology could not be diagnosed correctly until NGS was performed. This emphasizes the promising role of NGS in real practice, as a powerful tool of timely diagnosis instead of the long-lasting diagnostic odyssey of such patients.

## Data Availability Statement

The datasets presented in this study can be found in online repository: https://www.ncbi.nlm.nih.gov/sra/?term=SRR16916455.

## Ethics Statement

Ethical review and approval was not required for the study on human participants in accordance with the local legislation and institutional requirements. The patients/participants provided their written informed consent to participate in this study. Written informed consent was obtained from the individual(s) for the publication of any potentially identifiable images or data included in this article.

## Author Contributions

AL was treating the patient, performing her follow-ups, and writing this paper. AE was treating the patient, supervising all parts of this paper's preparation, and edited this paper. AM supervised the genetic testing of the patient and provided valuable comments on this case. AK performed the NGS and edited this paper. MD performed the Sanger sequencing. OD is the chief of the center who provided valuable comments on this case. MK performed the development of a method for the determination of plant sterols and performed determination of their concentration of phytosterols. All authors contributed to the article and approved the submitted version.

## Conflict of Interest

The authors declare that the research was conducted in the absence of any commercial or financial relationships that could be construed as a potential conflict of interest.

## Publisher's Note

All claims expressed in this article are solely those of the authors and do not necessarily represent those of their affiliated organizations, or those of the publisher, the editors and the reviewers. Any product that may be evaluated in this article, or claim that may be made by its manufacturer, is not guaranteed or endorsed by the publisher.
